# The effect of prehabilitation for older patients awaiting total hip replacement. A randomized controlled trial with long-term follow up

**DOI:** 10.1186/s12891-025-08468-4

**Published:** 2025-03-06

**Authors:** Odd-Einar Svinøy, Jakob Vangen Nordbø, Are Hugo Pripp, May Arna Risberg, Astrid Bergland, Pål Oliver Borgen, Gunvor Hilde

**Affiliations:** 1 Faculty of Health Sciences, Department of Rehabilitation Science and Health Technology, Oslo Metropolitan University, Oslo, Norway; 2 Department of Orthopedic Surgery, Akershus University Hospital, Lørenskog, Norway; 3 Institute of Clinical Medicine, Campus Ahus, University of Oslo, Oslo, Norway; 4 Oslo Centre of Biostatistics and Epidemiology, Research Support Services, Oslo University Hospital, Oslo, Norway; 5 Department of Sports Medicine, Norwegian School of Sport Sciences, Oslo, Norway; 6 Division of Orthopaedic Surgery, Department of Research, Oslo University Hospital, Oslo, Norway; 7 Department of Orthopedics, Martina Hansens Hospital, Bærum, Norway

**Keywords:** Hip arthritis, Prehabilitation, Gait speed, Elderly, Total hip replacement, Quality of life

## Abstract

**Background:**

Prehabilitation involving a planned exercise program before surgery is proposed to improve rehabilitation and postoperative outcomes. However, the current evidence on the efficacy of prehabilitation for patients awaiting total hip replacement is conflicting. The aim of this study was to evaluate efficacy of preoperative exercises and education (AktivA®) for adults 70 years or older awaiting total hip replacement.

**Methods:**

In a two-armed randomized controlled trial we recruited 98 participants aged 70 years or older with a Harris Hip Score less than 60 awaiting elective primary total hip replacement. Participants were recruited at three hospitals in Norway between 2019 and 2022. Participants were randomly assigned to prehabilitation or usual care. The prehabilitation group received a tailored exercise program for 6–12 weeks in addition to patient education. Gait speed, the primary outcome, was measured by the 40 m Fast-Paced Walk Test. Secondary outcomes included performance-based tests (Chair Stand Test, Timed Up & Go Test, 6-Minute Walk Test, Stair Climb Test) and patient-reported outcomes (Hip Disability and Osteoarthritis Outcome Score (HOOS) and EQ-5D). Outcomes were assessed at baseline, post intervention, and further 6 weeks, 3-, 6-, and 12 months post-surgery.

**Results:**

For the primary outcome gait speed at the primary endpoint (3 months post-surgery), no significant between-group differences were observed. However, post-intervention (before surgery), we found a significant improvement in favor of prehabilitation for both gait speed (0.15 m/s, 95% CI 0.02–0.28) and the HOOS quality of life subscale (11.93, 95% CI 3.38–20.48). No other significant differences were found at any post-surgery follow-up for these outcomes. For other secondary outcomes, there were no between-group differences at any point of assessment. Both groups showed improvement across all outcomes 3–12 months after surgery.

**Conclusions:**

The AktivA^®^program, used as a prehabilitation intervention during a period of 6–12 weeks before total hip replacement did not improve gait speed or any other post-operative outcomes compared to usual care. Both groups demonstrated significant improvement in gait speed and performed well relative to Western reference values 12 months post-surgery. Thus, replacing painful hip joints through total joint replacement seems to outweigh the efficacy of prehabilitation.

**Trial registration:**

ClinicalTrials.gov Identifier**:** NCT03602105—initial release: 06/06/2018.

**Supplementary Information:**

The online version contains supplementary material available at 10.1186/s12891-025-08468-4.

## Introduction

Osteoarthritis (OA) is one of the leading global causes of years lived with disability, with its prevalence increasing with age, and affecting approximately one in three adults above 70 years old [1]. Among weight bearing joints, hip OA is the second most prevalent after knee OA [1]. According to a meta-analysis by Fan et al. [2] the global pooled prevalence of hip OA, based on a Kellgren-Lawrence grade of ≥ 2, is estimated to 8.55% (95% CI 4.85 −13.18). Europe has the highest prevalence of hip OA with 12.59% (95% CI 7.17–19.25) [2]. Data from six European countries indicate that OA is strongly associated with frailty and prefrailty in the community dwelling individuals over the age of 65 [3]. OA is linked to poorer physical health and increased utilization of health care services [4, 5].

First-line treatment for hip OA consists of management programs that include exercise, patient education and weight reduction; key strategies for reducing pain and improving function [4–7]. However, when first-line treatment proves insufficient and the condition severely impacts the patient's quality of life, total hip replacement (THR) should be considered [7]. Worldwide, more than 1.4 million THRs are performed annually [8], with 10,812 recorded in Norway in 2023 [9]. Patients’ expectations for THR are high [10], and long-term expectations are often fulfilled [11–13], but not always [14]. Prospective studies indicate that 7–23% of the patients experience persistent long-term pain after having THR [14]. Pre-surgery factors such as reduced muscle strength, gait speed and balance are possible predictors of delayed recovery after total hip replacement [15]. A recent cross-sectional study using computed tomography found that the preoperative ratio of lean muscle mass / total muscle area may be negatively correlated with gait speed 6 months after THR [16].

Prehabilitation in terms of a planned exercise program before surgery is proposed to improve the rehabilitation process and postoperative outcomes [17]. Several systematic reviews have summarized the evidence on efficacy of exercise or exercise combined with patient education before THR, but they differ in their conclusions. Among the most recent reviews, Punnoose et al. [18] state moderate certainty evidence for improved muscle strength and improved quality of life after surgery in favor of prehabilitation prior to THR, whereas others find inconclusive evidence [19–21]. Some issues highlighted on efficacy of prehabilitation prior to THR include the use of low-to-moderate intensity exercise intervention and inclusion of participants who were not at high risk for delayed recovery [22–25].

The decline in physical performance with age is well documented [26, 27], with population studies showing a notable decrease after the age of 65 years [28, 29]. This decline is even more pronounced in individuals with OA [28, 30]. To focus on participants likely to benefit from prehabilitation, we chose to include patients aged 70 years or older with a Harris Hip Score less than 60, a score considered as poor [31] and often used by orthopedic surgeons as a criterion for severe hip OA, indicating eligibility for THR [32]. While there is no definitive conclusion regarding the effectiveness of prehabilitation for patients awaiting THR, further high-quality research with robust methodology and interventions is needed [22, 23, 33]. Additionally, more studies are required to specifically address older adults at higher risk for suboptimal surgical outcomes [25, 34].

The primary aim of this study was to assess the efficacy of a prehabilitation program consisting of exercises and education (AktivA®) [35] on postoperative gait speed for patients 70 years or older with Harris Hip Score < 60 awaiting THR. Secondary aims were to assess the efficacy of prehabilitation on additional outcomes including pain, symptoms, activity of daily living (ADL), physical activity, and quality of life (QoL). Performance-based tests such as walking long distances, getting in and out of a chair, ambulatory transitions and walking up and down stairs [36] were also assessed as secondary outcomes. The exercise intervention was designed to meet the guidelines set by the American College of Sports Medicine [37].

## Methods

### Design and setting of the study

This study was a two-armed randomized controlled trial, comparing the efficacy of a prehabilitation program (AktivA®) against usual care on performance-based physical outcomes and patient reported outcome measures. The study protocol has been previously published [38]. The study was conducted in South-Eastern Norway, and participants were included from Akershus University Hospital, Martina Hansens Hospital and Diakonhjemmet Hospital between November 2019 and December 2022. The orthopedic surgeons at the collaborating hospitals screened for eligible participants meeting the study's inclusion criteria and assessed the patient’s severity of hip pain and functional limitations using the Harris Hip Score. Physiotherapists delivered the prehabilitation before surgery in a real-world clinical setting in the primary health care sector. All physiotherapists delivering the intervention had attended an 8-h AktivA course, as part of a national model for implementation of evidence-based guidelines for patients with OA [35].

The completed study had some deviations from the published study protocol [38]. While we initially planned to include 150 participants, slow recruitment rates and challenges posed by the Covid-19 pandemic resulted in the inclusion of 98 participants. For participants whose surgery dates were rescheduled and delayed beyond 12 weeks, the intervention period was continued until surgery. The primary endpoint of interest was set to 3 months post-surgery [38]. After obtaining additional funding for long-term follow-ups, we have also included follow-up assessment at 6- and 12-months post-surgery to the current study.

All physiotherapists delivering the prehabilitation intervention had access to appropriate exercise facilities at their respective clinics. The study adheres to the CONSORT checklist for randomized controlled trials [39] and the CHAMP statement [40].

### Study population

We included community-dwelling participants aged 70 years or older with residential addresses in the county of Oslo or Akershus scheduled for elective primary THR due to end-stage osteoarthritis. Additional inclusion criteria included a Harris Hip Score of less than 60 [31, 41], mental capability to follow the preoperative program, and being able to read and fill out questionnaires independently. Participants were excluded if they had known rheumatoid arthritis, medical contraindications for physical activity, neurological disease affecting gait or were unable to speak and understand the Norwegian language. Furthermore, we excluded participants already enrolled in an AktivA® program.

### Randomization and blinding

Participants were randomly allocated to the intervention or control group with a 1:1 ratio. A computer-generated random number sequence with randomly permuted block sizes and opaque sealed envelopes was used for group allocation. The randomization to groups was performed by the researcher coordinating the study. This person was not involved in the recruitment of study participants and did not perform outcome assessments at any point in time throughout the study. Participants were stratified by the hospital performing the surgery. Outcome assessors and research personnel entering data into the data files were blinded to group allocation.

### Intervention

The exercise intervention was described following the Consensus on Exercise Reporting Template (CERT) [42] (Appendix File 1). The prehabilitation program included exercises and patient education [35] lasting 6 to 12 weeks. Participants completed 3 to 4 weekly training sessions, each lasting 45 to 60 min. Two of the weekly sessions were supervised by an experienced physiotherapist individually, or in a group. The remaining session(s) were performed at home following a prescribed exercise program provided by the supervising physiotherapist. The exercise program included both progressive resistance training and neuromuscular training, tailored to each participant's specific needs, with a focus on large muscle groups. Key exercises included leg presses, leg extensions, gluteal bridges, and functional movements such as squats, lunges, and balance exercises [38].

General recommendations for exercise dosage and progression were followed [37, 43], with resistance exercises targeting 40–60% of one repetition maximum, allowing for 8–12 repetitions in 1–3 sets. Resistance equipment, including bands, dumbbells, and machines, was used to tailor and adjust the resistance level for each individual. Load and progression were adjusted based on pain levels; if participants rated their pain as 5 or higher on a 0–10 Numeric Rating Scale after exercise, modifications to the exercise program and dosage were made. The physiotherapist tracked adherence to the supervised sessions, while participants self-recorded their adherence to home-based training using exercise diaries. Overall adherence was defined as completing 80% or more of both the supervised and home-based training sessions. The education component of the intervention provided patients with information on arthritis management, the importance of physical activity, and when applicable, recommendations for weight loss. Education was delivered by the physiotherapist either through group sessions or personalized guidance.

Participants in the control group received standard care without supervised prehabilitation before surgery. Regardless of group, all participants received standard preoperative information and preparation from the recruiting hospital.

### Assessment and outcome measures

Outcome assessments were performed at baseline (before randomization), post intervention (within a week after ended intervention), and 6 weeks and further 3-, 6- and 12 months after THR surgery. Gait speed measured by the 40 m Fast Paced Walk Test [36] was the primary outcome, and the primary endpoint set to 3 months post-surgery. Secondary performance-based outcomes included Chair Stand Test [44], Timed Up & Go Test [45], 6 Minute Walk Test (6 MWT) [46], and Stair Climb Test [47]. Additional self-reported secondary outcomes were the Hip Disability and Osteoarthritis Outcome Score (HOOS) with subscales for pain, symptoms, activity of daily living (ADL), physical activity, and quality of life [48] and the and the EQ visual analogue scale (EQ VAS) rating general health [49]. Sociodemographic data and use of health care services were collected through questionnaires. Testing was administered by the researcher coordinating the study in collaboration with outcome assessors blinded to group allocation. The outcome assessors were all experienced physiotherapists employed as research assistants in the project or they worked at the collaborating hospital paid by the hour. Before project start, they were trained in the testing procedures for all the performance-based outcomes in our study, using the standardized procedures outlined by the Osteoarthritis Research Society International [36], except for the 6 MWT. Due to practical reasons, the 6 MWT was performed by walking back and forth along a 15-m straight line covering as much ground as possible over 6 min. Performing the 6 MWT by walking back and forth has shown acceptable reliability and construct validity [50].

### Sample size

Sample size estimation was based on a clinically meaningful between-group difference in gait speed at the primary end point 3 months post-surgery. As described in our protocol paper [38], a substantial meaningful mean difference between groups at 3 months post-surgery in habitual gait speed was set at 0.1 m/s with an expected standard deviation (SD) of 0.2 m/s. This estimate was based on findings by Perera et al. [51]. The sample size calculation was performed using an analysis of covariance (ANCOVA) model to assess the mean difference between the randomized groups. Based on this calculation a sample size of 120 participants was sufficient to obtain 80% statistical power. The recruitment rate was substantially slowed down due to the Covid-19 pandemic and a recalculation of the sample size was performed to potentially reduce the required study sample size. Based on the recalculation, we included 98 participants instead of 120, which reduced the statistical power by less than 10%. However, by using the Stata command sampsi, the recalculation still ensured 80% statistical power with an ANCOVA model, assuming a correlation of 0.5 between the baseline and follow-up measurement.

### Statistical methods

Our statistical analysis plan outlining the analytical approach for data gathered in this study was published at ClinicalTrials.gov [52] before we revealed the group allocation variable to our data files ready to be analyzed. Demographic and baseline characteristics in the two study groups were presented as number (n) and percentage (%) for categorical data, and for continuous data by mean and standard deviation (SD) or median with minimum and maximum values (min–max) as appropriate depending on the distribution of the variable. Within group analyses were explored using the Paired-samples t-test if data were normally distributed, if not the Wilcoxon Signed Rank test was used.

Linear mixed models for repeated measurements were our primary analysis of differences between randomized groups for continuous outcome variables. We assessed mean differences in primary outcome between randomized groups at each follow-up time with 95% CI and p-value using estimated marginal means from the maximum likelihood estimated models. The model included a random intercept to account for the within subject correlation of repeated measurements and the following independent fixed effects: the respective outcome variable at baseline, the follow-up times, the randomized groups, the interaction term between randomized groups and follow up times. Secondary outcomes were assessed using statistical procedures similar to the primary outcome. Intention to treat (ITT) was the principal analysis assessing effect of the intervention. We also performed a ‘per-protocol analysis’ including those completing the trial with an overall exercise adherence ≥ 80%.

All calculated *p*-values were two-sided and set to a 5% significance level. StataSE 18.0 for Windows (StataCorp LLC, 4905 Lakeway Drive, College Station, TX, 77,845, USA) were used to conduct statistical analysis.

To assess the impact of missing data during follow-up, we explored the missing data mechanism by comparing participants with or without missing data at any point of follow-up assessment against their respective baseline values. We assumed the missing data to be either completely at random (MCAR) or missing at random (MAR) and applied multiple imputation (MI) to assess the robustness of our dataset in terms of precision or variance, accuracy, and power [53]. In case the missing data were not at random (MNAR), we also used MI as a sensitivity analysis to explore the robustness of our analysis [53]. In building our model for imputation we conducted 25 multiple imputations using chained equations and linear regressions models. These were based on data from baseline to end of follow-up for the respective outcome, and baseline data on age, sex, TUG, and HOOS quality of life. For imputation of TUG and HOOS quality of life, we used baseline data of the gait speed measured with the 40 m Fast Paced Walk Test. We examined the distribution of the imputed data for the outcome measure to ensure the values that were realistic. Any values outside the lower or upper range of observed values were replaced with the observed minimum or maximum value respectively.

### Ethical considerations

All participants signed an informed consent before the baseline assessment. The Regional Ethical Committee in the Health Region South-East in Norway approved the study protocol (ref no. 2018/503), and the Data Inspectorate at the collaborating hospitals approved the study.

## Results

**Fig. 1:**
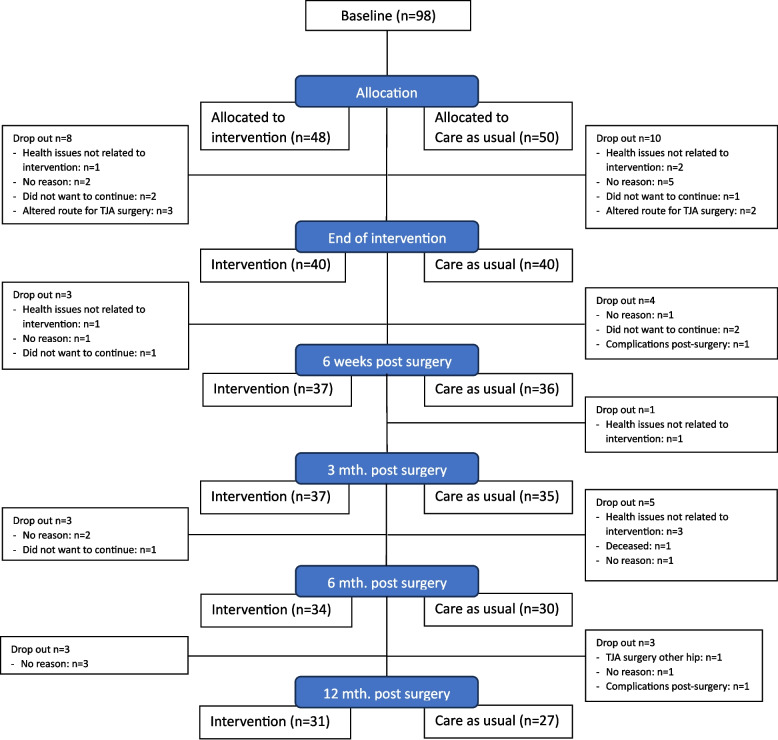
Participant flow-chart

We ceased participant enrollment at 98 individuals, assigning 48 to the intervention group and 50 to the control group through randomization. At the primary endpoint, 3 months post-surgery, 26 participants (27%) had withdrawn their consent. This withdrawal rate increased to 34% at 6 months, and further to 40% at 12 months post-surgery. The dropout rates were comparable between the intervention and control groups. The flow-chart (Fig. 1) provides information on participant dropout and reasons for withdrawal. Additionally, we encountered missing data primarily due to the Covid-19 pandemic, during which outpatient clinics at the hospitals were closed for periods, and many participants were hesitant to attend in-person testing after reopening. Also with reopened outpatient clinics, some participants did not attend scheduled follow-ups despite repeated reminders. We analyzed the missing data and determined that the missing mechanism was mostly missing completely at random or missing at random for follow-ups until 3 months, but not missing at random at 6- and 12-months post-surgery. Notably, control group participants with poor baseline gait speed performance were more likely to be lost to follow-up at six and twelve months compared to those in the intervention group.

The mean age was 76.8 ± 4.4 years in the intervention group and 76.3 ± 4.7 years in the control group. For Harris Hip Score, the intervention group had an average score of 48.02 ± 9.73 and the control group 47.64 ± 7.37. There were no significant differences between the groups in baseline characteristics, except for body height, where participants in the intervention group were somewhat taller than those in the control group. Baseline characteristics of the participants are provided in Table 1. The median length of the intervention was 11 weeks (range 4 to 20 weeks). Two prehabilitation participants had an intervention period of 20 weeks, as their THR surgery was rescheduled by several weeks. Apart from the prehabilitation, the comparison groups did not differ in their use of healthcare services during the intervention period. Additionally, there were no differences between the groups in their use of physiotherapy or other healthcare services during the postoperative period. Three months post-surgery, the intervention group reported an average of 8.26 physiotherapy sessions, while the control group reported 6.34 sessions over the previous 12 weeks.

**Table 1:** Baseline characteristics of study participants

Characteristics	Intervention (*n* = 48)	Control (*n* = 50)
Age (years)	76.84 ± 4.40	76.30 ± 4.73
Female (%)	27 (56%)	37 (74%)
Weight (kg)	79.62 ± 17.21	79.02 ± 14.19
Height (cm)*	169.54 ± 11.22	167.80 ± 8.39
BMI (kg/m^2^)	27.56 ± 4.60	28.10 ± 4.95
Level of education		
- Primary	18 (37.5%)	13 (26.5%)
- Secondary	12 (25.0%)	20 (40%)
- Tertiary	17 (35.4%)	17 (34%)
- Missing	1 (2.1%)	-
Harris Hip Score (0–100)	48.02 ± 9.73	47.64 ± 7.37
ASA-score: n (%)		
- ASA 1	-	2 (4%)
- ASA 2	34 (71%)	31 (62%)
- ASA 3	14 (29%)	15 (30%)
- Missing ASA	-	2 (4%)
FCI (numbers of comorbidities)	1.40 ± 1.29	1.44 ± 1.48
EQ-VAS (0–100)	56.55 ± 21.64	54.00 ± 17.52
**Physical outcome measures**		
- 40 m Fast-Paced Walk Test (m/s)	1.21 ± 0.38	1.29 ± 0.37
- 30 s Sit-to-Stand Test (no. of repetitions)	10.33 ± 3.71	10.02 ± 3.64
- Timed Up and Go Test (s)	11.13 ± 3.41	11.13 ± 3.63
- 6 min Walk Test (m)	353.04 ± 104.98	353.23 ± 104.12
- Stair Climb Test (s)	18.51 ± 8.41	19.99 ± 11.78
**HOOS (0–100)**		
- Pain	41.33 ± 12.05	41.65 ± 12.57
- Symptoms	41.41 ± 16.72	39.08 ± 14.67
- ADL	42.08 ± 13.41	44.53 ± 14.71
- Sports/recreation	26.85 ± 19.46	27.00 ± 17.74
- QOL	27.13 ± 13.74	26.5 ± 13.86

Continuous variables given as means (± standard deviation, SD), categorical variables as numbers (percentages, %)

Tests of statistical significance were done with independent samples t-tests for continuous variables and chi-squared tests for categorical variables

*HOOS* Hip Disability and Osteoarthritis Outcome Score, *ADL* activities of daily living, *QOL* quality of life, *ASA-score* American Society of Anesthesiologists Physical Status Classification System, *FCI* Functional comorbidity index, *EQ-VAS* EuroQol visual analogue scale; part of the EuroQol 5-dimensional quality of life questionnaire

^*^Statistically significant difference between groups (*p* < 0.05)

### Between-group differences for primary outcome

For the primary outcome gait speed, no significant between-groups differences were found at the primary endpoint 3 months post-surgery (mean diff 0.08 m/s; 95% CI: −0.06 to 0.21) or at any other points of assessment post-surgery. However, post-intervention (before surgery), we observed a significant between-group difference in favor of prehabilitation for gait speed (mean diff 0.15 m/s; 95% CI: 0.02 to 0.28). After applying multiple imputation for missing participant data, the between-group difference for gait speed remained consistent, indicating no effect of the prehabilitation intervention neither at 3 months after surgery nor at any other post-surgery follow ups (Table 2). We also performed a sensitivity analysis for gait speed where we adjusted for differences in body height and sex at baseline, the analysis yielded similar results as the primary analysis. The per-protocol analysis also yields results similar to the primary findings (Appendix Table 3).

**Table 2:** Mean differences between the intervention and control group (95% CI)

Outcome	Intention to treat: Intervention vs. control		Multiple imputation for MPD: Intervention vs. control	
	Mean difference	95% CI	Mean difference	95% CI
40 m Fast-Paced Walk Test (m/s)				
Post-intervention	0.15*	0.02, 0.28	0.20*	0.02, 0.36
6 weeks post-surgery	−0.01	−0.16, 0.13	−0.01	−0.29, 0.28
3 months post-surgery	0.08	−0.06, 0.21	0.07	−0.09, 0.24
6 months post-surgery	0.01	−0.13, 0.14	0.02	−0.14, 0.18
12 months post-surgery	0.11	−0.03, 0.25	0.14	−0.03, 0.32
30 s Sit-To-Stand Test (no. of rep.)				
Post-intervention	1.16	−0.19, 2.52	1.57	−0.32, 3.46
6 weeks post-surgery	−0.11	−1.69, 1.48	−0.32	−2.68, 2.04
3 months post-surgery	0.80	−0.56, 2.16	0.95	−0.90, 2.79
6 months post-surgery	0.81	−0.56, 2.19	0.40	−1.58, 2.38
12 months post-surgery	1.37	−0.04, 2.78	1.48	−0.82, 3.77
Timed Up and Go Test (s)				
Post-intervention	−0.76	−1.54, 0.01	−0.59	−1.73, 0.55
6 weeks post-surgery	0.70	−1.12, 1.71	0.76	−0.96, 2.48
3 months post-surgery	−0.14	−0.92, 0.63	−0.14	−1.11, 0.83
6 months post-surgery	0.12	−0.66, 0.91	0.38	−0.66, 1.42
12 months post-surgery	−0.23	−1.05, 0.57	0.04	−0.88, 0.97
6 min Walk Test (m)				
Post-intervention	18.98	−15.02, 52.99	31.94	−14.63, 78.52
6 weeks post-surgery	26.77	−10.56, 64.10	35.51	−26.44, 97.47
3 months post-surgery	13.52	−20.44, 47.48	11.13	−33.04, 55.30
6 months post-surgery	27.84	−5.50, 62.08	32.23	−10.65, 75.11
12 months post-surgery	19.21	−15.67, 54.09	15.95	−28.68, 60.59
Stair Climb Test (s)				
Post-intervention	−0.86	−3.36, 1.63	−0.92	−4.60, 2.75
6 weeks post-surgery	−0.38	−3.23, 2.47	1.00	−5.69, 7.70
3 months post-surgery	−0.26	−2.71, 2.18	−0.34	−3.45, 2.76
6 months post-surgery	0.77	−1.75, 3.29	2.15	−0.62, 4.93
12 months post-surgery	−0.53	−3.16, 2.10	−0.18	−3.24, 2.88
HOOS Pain (0–100)				
Post-intervention	2.06	−5.20, 9.32	1.90	−5.37, 9.18
6 weeks post-surgery	0.29	−7.32, 7.92	0.91	−13.22, 15.05
3 months post-surgery	−1.20	−7.92, 5.40	−1.34	−8.36, 5.68
6 months post-surgery	−2.65	−9.72, 4.42	−3.09	−11.03, 4.86
12 months post-surgery	−3.03	−10.35, 4.29	−4.07	−12.99, 4.83
HOOS Symptoms (0–100)				
Post-intervention	4.69	−2.59, 11.98	3.06	−6.37, 12.49
6 weeks post-surgery	−1.94	−9.51, 5.62	−2.37	−11.60, 6.86
3 months post-surgery	−2.39	−9.15, 4.41	−3.56	−11.85, 4.74
6 months post-surgery	−3.13	−10.16, 3.90	−1.86	−9.49, 5.67
12 months post-surgery	−3.84	−11.34, 3.66	−6.10	−13.96, 1.75
HOOS ADL (0–100)				
Post-intervention	8.66	−1.84, 19.16	2.51	−6.38, 11.40
6 weeks post-surgery	4.20	−7.69, 16.10	−0.83	−9.66, 8.00
3 months post-surgery	−2.40	−12.45, 7.63	−2.61	−9.80, 4.58
6 months post-surgery	1.95	−8.43, 12.33	−1.34	−9.20, 6.52
12 months post-surgery	−7.15	−18.09, 3.78	−3.92	−11.29, 3.44
HOOS Sports/recreation (0–100)				
Post-intervention	8.66	−1.84, 19.16	5.26	−5.60, 16.14
6 weeks post-surgery	4.20	−7.69, 16.10	1.51	−24.29, 21.28
3 months post-surgery	−2.40	−12.45, 7.63	−0.30	−12.74, 12.14
6 months post-surgery	1.95	−8.43, 12.33	6.01	−7.16, 19.20
12 months post-surgery	−7.15	−18.09, 3.78	−3.80	−17.90, 10.30
HOOS QoL (0–100)				
Post-intervention	11.93*	3.38, 20.48	9.89*	0.77, 19.01
6 weeks post-surgery	4.79	−4.27, 13.85	1.32	−13.04, 15.67
3 months post-surgery	−4.86	−12.85, 3.12	−2.94	−13.28, 7.40
6 months post-surgery	−1.29	−9.63, 7.05	−2.93	−11.30, 5.45
12 months post-surgery	−5.03	−13.80, 3.74	−6.66	−15.84, 5.52
EQ-VAS (0–100)				
Post-intervention	7.19	−0.34, 14.73	6.68	−3.00, 16.36
6 weeks post-surgery	−7.61	−15.52, 0.28	−8.17	−19.02, 2.69
3 months post-surgery	−2.64	−9.63, 4.35	−3.28	−11.87, 5.31
6 months post-surgery	1.21	−6.04, 8.46	2.12	−7.07, 11.32
12 months post-surgery	2.30	−5.44, 10.05	1.27	−8.56, 11.11

Differences between randomized groups for continuous outcome variables analyzed by linear mixed models for repeated measurements, p-values were two-sided and set to a 5% significance level but not presented in the table, *statistical significance between groups *p* < 0.05

*CI* Confidence interval, *MPD* missing participant data, *HOOS* Hip Disability and Osteoarthritis Outcome Score, *ADL* activities of daily living, *QOL* quality of life, *EQ-VAS* EuroQol visual analogue scale; part of the EuroQol 5-dimensional quality of life questionnaire

### Between-group differences for secondary outcomes

Post-intervention, the mean difference between groups for the HOOS quality of life subscale was 11.93 in favor of the intervention group (95% CI: 3.38 to 20.48). However, there were no significant between-group differences for this outcome at the primary endpoint 3 months post-surgery or any other follow ups. For other secondary outcomes we found no between-group differences at any point of assessment. Analysis after imputation for missing data yielded consistent results (Table 2). The per protocol analysis of secondary outcomes produced similar findings, except from the 30 s Sit-to-Stand test. The mean differences in number of repetitions in Sit-to-Stand were 1.81 repetitions (95% CI: 0.01 to 3.60) in favor of the intervention group 3 months post-surgery and 1.89 repetitions (95% CI: 0.14 to 3.69) 12 months post-surgery (Appendix Table 3).

### Within group differences between baseline and follow-up

**Fig. 2:**
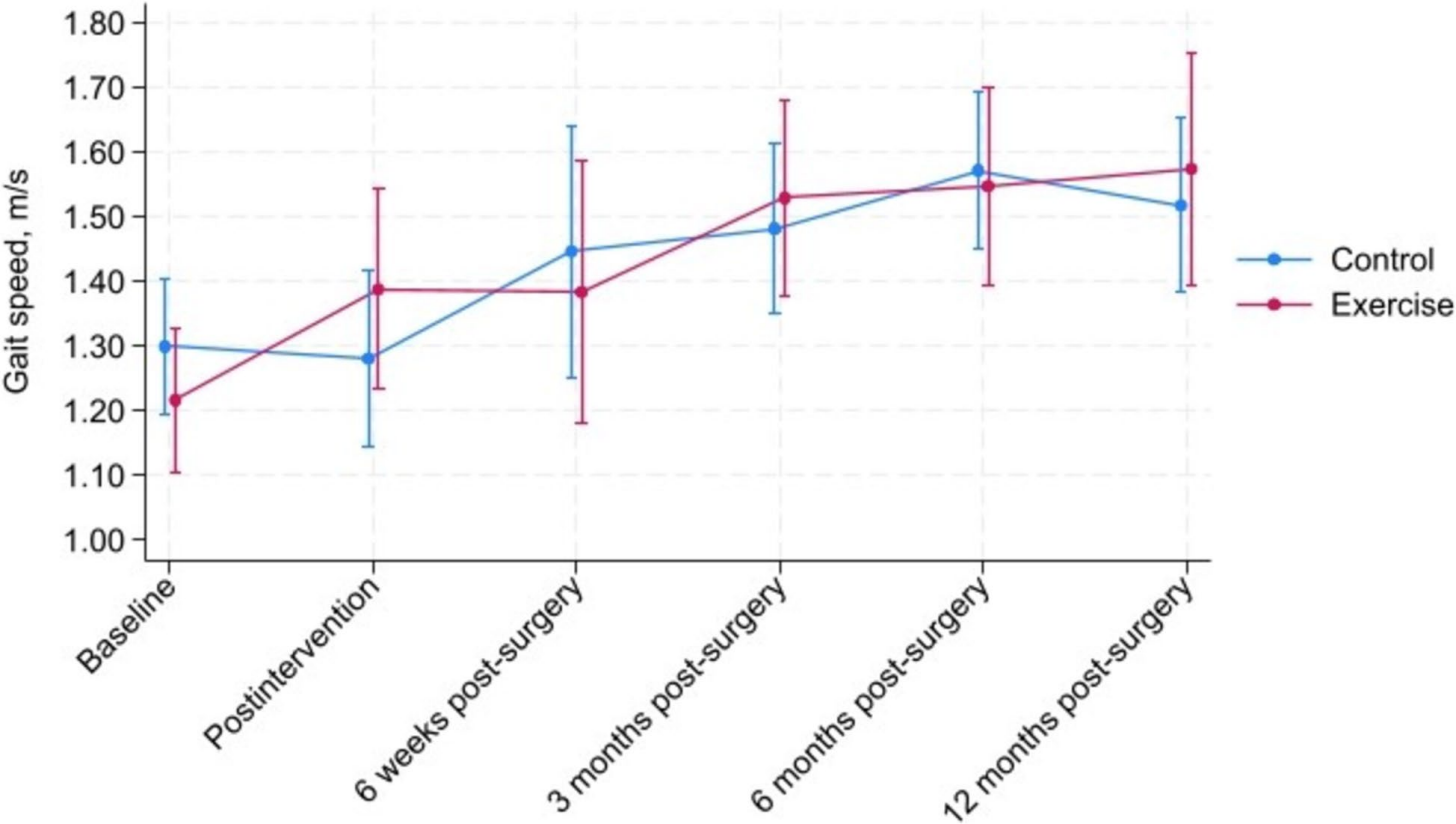
Intention to treat analyses with point estimates on the primary outcome measure gait speed at all points of measurement within the prehabilitation group and control croup respectively

Both groups demonstrated improvement across all outcomes 3–12 months after surgery. For the primary outcome, gait speed, the mean change in score between baseline and 3 months after surgery was 0.31 m/s (p < 0.05) and 0.19 m/s (p < 0.05) within the intervention and control group respectively. At 12 months post-surgery, the improvement relative to baseline was 0.36 m/s (p < 0.05) within the intervention group and 0.22 m/s (p < 0.05) within the control group. The improvements in gait speed within the groups throughout the study period are also displayed in Fig. 2 (ITT-analysis). Appendix Table 4 shows descriptive values and number of observations of all outcome measures within each group at all assessment points. Physical performance scores, stratified by sex, are presented in Appendix Table 5.

### Adherence

Among the prehabilitation participants who did not drop out but completed their intervention period, 34 out of 40 (85%) were adherent to the supervised exercise protocol, whereas 31 out of 40 (77%) were adherent to the unsupervised exercise protocol.

### Use of aids during physical testing

At the post-intervention assessment, the control group showed significantly higher use of walking aids when performing the TUG test (p < 0.05). No other significant between-group differences were observed in the using any aid at any time point. Frequencies of aid usage during physical performance testing are presented in Appendix Table 6.

### Adverse events

No preoperative adverse events linked to the study intervention, or the clinical assessments, were recorded throughout the study period. However, one participant in the control group experienced a post-surgical complication involving a neurological disorder of the foot and subsequently withdrew from the study 6 months after surgery.

## Discussion

No significant between-group differences in gait speed were observed at the primary endpoint 3-month post-surgery or any other post-surgery assessments. This indicates that the prehabilitation program did not affect gait speed postoperatively in community-dwelling participants aged 70 years or older who were scheduled for elective primary THR due to end-stage osteoarthritis. Similarly, no significant differences were found for any secondary outcomes at any post-surgery time point.

As the exercise intervention duration in our study ranged from 4–20 weeks (median 11 weeks), most participants met our study protocol outlining an intervention period of 6 to 12 weeks [38]. An exercise program exceeding 12 weeks could potentially enhance the efficacy of our exercise program. However, ethical requirements to avoid delaying surgery, extending the intervention period was not feasible in our study. Progression of exercise dosage through the training period is crucial for musculoskeletal and cardiovascular adaptations [54]. Prior studies have shown that patients awaiting THR can tolerate progressive resistance training without adverse effects [33], and that moderate- [55] to high-intensity [56] training is feasible. Although our prehabilitation intervention intended to follow current exercise recommendations for dose and progression [37, 43], the physiotherapists delivering the intervention reported through verbal communication that progression was particularly challenging for our study participants as they experienced significant pain during and after exercise. The supervising physiotherapists had to balance intensity with participant willingness to continue exercising throughout the intervention period, sometimes resulting in a lower exercise intensity than prescribed in the study protocol [38]. This may explain the limited post-surgery efficacy of prehabilitation in our study. Another factor that could influence the limited effect on gait speed is the lack of focus on gait-training exercises in our prehabilitation program.

The effectiveness of an exercise program relies heavily on adherence to the exercise protocol, making it a crucial predictor of outcomes [57]. In our study, satisfactory adherence was defined as attending at least 80% of the scheduled sessions, a common threshold in trials [58]. Eighty-five percent of the prehabilitation participants attended 80% or more of the planned supervised sessions. Given the age of the study sample, the use of outreach-supervised training at home could potentially have improved adherence even more [59]. However, while outreach visits by physiotherapists delivering the intervention were an option in our study, none of the participants opted for outreach visits. For the unsupervised home sessions, 77% completed at least 80% of the sessions, which was lower than expected, though not uncommon. A systematic review by Smith et al. [60] reported an average adherence rate of 67.9% for unsupervised exercise, which is based on data from 72 trials examining exercise interventions for patients with osteoarthritis.

To our knowledge, only three previous RCTs have evaluated gait speed (m/s) for prehabilitation before THR [61–63]. Unlike our findings, Wang et al. [62] reported no prehabilitation effect before surgery but a significant effect post-surgery at 3 weeks, 3 months, and 6 months. In line with our study, Villadsen et al. [61] found no difference between groups at 6 weeks or 3 months post-surgery, whereas Holsgaard-Larsen et al. [63] found an effect at 3 months post-surgery with a mean difference of 1.5 m/s (95% CI: 0.2 to 2.7), though the difference was not significant at 12 months.

Four previous RCTs on prehabilitation before THR assessed gait capacity using the 6 MWT [55, 59, 62, 64]. Two studies [59, 64] found significant improvements in the 6 MWT before surgery, while our results showed no effect. Post-surgery, former studies report in line with our findings no significant differences between comparison groups neither at short term [55, 59] nor when assessed 3- and 6 months post-surgery [62].

Six former RCT publications report findings on mobility measured by the TUG test [55, 59, 61, 64–66]. Apart from Zeng et al. [64] who reported improved TUG scores before surgery, no other studies found significant differences between comparison groups either before or after surgery [55, 59, 65]. These findings are consistent with our study.

Eight previous RCTs [55, 56, 59, 61, 63, 65, 67, 68] have assessed lower extremity strength. Three of these studies [55, 59, 61] reported no significant differences between comparison groups neither before nor after surgery. Contrary to our findings, four studies [56, 65, 67, 68] reported enhanced muscular strength favoring prehabilitation before surgery and two studies [63, 67] demonstrated an effect of prehabilitation program on postoperative function.

We found a significant effect of the prehabilitation program on the HOOS quality of life subscale post-intervention. This aligns with Hermann et al. [56] but contrasts with other RCTs [55, 59, 61]. However, we found no significant between-group differences for any of the other patient-reported outcomes, consistent with five other RCTs [55, 59, 61, 68, 69]. Holsgaard-Larsen et al. [63] found significant effects on the HOOS sport/recreation subscale at 3 months post-surgery but not at later follow-ups.

### Heterogeneity

The descriptive comparison of study results above is complicated by heterogeneity across studies. Gilbey et al. [67] concluded that customized prehabilitation exercises before THR were effective in enhancing functional recovery after surgery. This conclusion is based on their significant findings favoring the intervention group both at short term and long term (6 months). However, the participants in their intervention group also engaged in supervised and homebased exercise from 3–12 weeks post-surgery making it difficult to isolate the effect of prehabilitation during post-surgery follow ups. This limitation also applies to Wang et al. [62], as this study is a sub study of the RCT presented by Gilbey et al. Moreover, previous studies differ in participants characteristics. The mean age in our study was 76 and 77 years in the intervention- and control group respectively, comparable to the study samples included by Hoogeboom et al. [55] and Oosting et al. [59]. In contrast, the other studies referred to above included younger participants. Additionally, some of these studies have small sample sizes [55, 59, 62, 68], making them vulnerable for type II error [70]. However, two of these studies were designed as pilot RCTs [55, 59] and were not specifically aimed at detecting possible differences between groups. Furthermore, the studies varied in the testing modalities used to assess outcomes, complicating direct comparisons. An example is gait speed, measured as habitual gait speed in the study by Wang et al. [62], while Villadsen et al. [61] and Holsgaard-Larsen et al. [63] reported both habitual and fast paced gait speed. Similarly, lower extremity strength was measured using different methods, such as 1-repetition maximum testing [56, 63, 65, 67], functional testing [55, 59, 61] and subjective grading [68].

### Strengths and limitations

A strength of this study is that the physiotherapists delivering the intervention were experienced in working with this specific patient population. All physiotherapists had completed an 8-h AktivA®-course, part of a national model designed to implement evidence-based practice for patients with OA [35]. This study was conducted in a real-world clinical setting, utilizing outcome measures that are manageable in practice, which enhance the external validity of the findings. Additionally, the study population was comparable to the age-matched THR population at the participating hospitals during the recruitment period based on age, sex, and ASA-score [71, 72], highlighting the high external validity of the study. We followed the participants for 12 months after surgery, addressing a common shortcoming in many RCTs including short-term follow-up assessments [73]. An added strength of the study is that the outcome assessors were kept blinded to group allocation, and utilization of valid and reliable performance-based outcome measures relevant to the intervention’s end users [36].

The study has several limitations. The high dropout rate and missing participant data limits the interpretation of the results. In line with several ongoing RCTs during the Covid-19 pandemic we had considerable challenges in including participants. During the early stages of the pandemic, participant testing was not possible due to the closure of outpatient facilities. When these facilities reopened, many participants hesitated to meet in crowded areas, such as public hospitals, contributing to continued missing data. Before all testing appointments, participants received phone calls and text messages to confirm their attendance. For those who did not show up for testing, the project coordinator and physiotherapists called and sent them text messages to reschedule the appointment. Unfortunately, many did not respond or chose not to attend the testing appointment.

Missing participant data may affect the precision of the between-group differences and increase the risk of type II error due to lack of statistical power [53]. The MNAR missing mechanism for missing data at 6- and 12-months post-surgery may also lead to type II error, as control participants with poor baseline performance in gait speed were more likely to be lost to follow-up, potentially underestimating a possibly treatment effect. Nevertheless, after applying MI to address the missing data, the between-group difference in gait speed remained consistent with our primary analyses without MI, thus lending credibility to our findings, despite the high rate of missing data. We chose to replace missing data through MI, as simulation studies have shown that MI performs well even when the missing mechanism is assumed to be MNAR [53].

Measures to avoid missing participant data are important in clinical trials. In our study, the project coordinator was employed in an academic position at the university and not in a clinical position at the recruiting hospitals, and the General Data Protection Regulation (GDPR) privacy restrictions limited the project coordinator contacting participants until they gave informed consent. Consequently, the initial contact between the study participant and the project coordinator was made through a phone call rather than an in-person meeting. Earlier closer contact between the project coordinator and the participants might have increased willingness to attend testing and reduced drop-out rates throughout the study. Another limitation of the study is the absence of complete exercise diaries from intervention participants. While some training diaries were thoroughly completed, providing valuable data on intensity and progression, many were incomplete, which limited our ability to draw definitive conclusions about the dose–response relationship.

### The postoperative course

Improvements were observed in both groups across all outcomes at the follow-ups after surgery. For gait speed at baseline, the men in the intervention group scored on average 1.25 m/s and the control group 1.48 m/s, and for the women scored 1.19 m/s and 1.23 m/s respectively. Reference values on maximal gait speed from Denmark [74] and the United States [75], show a score of 2.01 m/s and 2.07 m/s respectively for men in their 70 s and 1.81 m/s and 1.74 m/s respectively for women in their 70 s. This demonstrates that our study sample had considerably lower walking speed at baseline when compared to reference values in healthy populations from Denmark and the US. However, 12 months post-surgery the mean gait speed among men in our study were 1.88 m/s and 1.73 m/s in intervention- and control-group respectively, whereas the corresponding scores for women were 1.44 m/s and 1.46 m/s, thus closing the gap to the reference values reported by Tibaek [74] and Bohannon [75].

For the secondary outcome measure, TUG, population-based reference values from Norway are available [28]. At baseline our male participants scored 10.68 s and 10.22 s in the intervention and control group respectively, a score that positioned them close to the reference value presenting the 75th percentile (10.7 s) for the male population aged 75 years. Corresponding baseline values for women were 11.49 s and 11.45 s, also representing scores close to the 75th percentile (10.9 s) for the female population aged 75 years. At 12 months post-surgery, a significant improvement was observed for both sexes. Men in the intervention- and control group scored 7.62 s and 7.35 s respectively, positioning both groups close to the 10th percentile (7.2 s), whereas women scored 8.70 s and 8.69 s respectively, thus close to the 25th percentile (8.2 s). These results highlight significant mobility gains after THR regardless of the pre-operative group allocation.

### Clinical implications

The efficacy of prehabilitation was not seen in any post-surgery measurements in our study. However, having a THR had an immense effect on all measured outcomes both for intervention and control participants. Notably, both groups performed well against Western reference values on fast-paced gait speed 12 months post-surgery. Therefore, this older population should be prioritized for early surgery and rehabilitation rather than spending time in prehabilitation. Importantly, although our findings show that prehabilitation did not directly improve postoperative outcomes, there may be potential for prehabilitation to prevent functional deterioration for patients facing extended waiting times before their total hip replacement. Further investigations are warranted.

## Conclusion

The AktivA® program used as a prehabilitation intervention before THR was evaluated against usual care, and did not result in improvement in the primary outcome gait speed or improvement in any of the secondary outcomes, neither at the primary endpoint of 3 months post-surgery or at any other post-surgery assessments. Both groups showed improvement across all outcomes 3–12 months after surgery and performed well against reference values for gait speed at 12 months post-surgery. This suggests that the benefits of replacing painful hip joints through TJR outweigh the impact of this prehabilitation program. The study had limitations, including dropouts, missing participant data, and challenges with exercise progression.

## Supplementary Information

Supplementary Material 1.

Supplementary Material 2.

Supplementary Material 3.

Supplementary Material 4.

Supplementary Material 5.

Supplementary Material 6.

## Data Availability

Data may be available for replication analysis in an anonymous format in accordance with GDPR. For request, contact the corresponding author.

## Abbreviations

OA: Osteoarthritis
CI: Confidence Interval
THR: Total Hip Replacement
QoL: Quality of Life
RCT: Randomized Controlled Trial
PROMs: Patient Reported Outcome Measures
CONSORT: Consolidated Standards of Reporting Trials
CHAMP: Consensus on Hip Arthroplasty Measurement Properties
MCAR: Missing Completely at Random
MAR: Missing at Random
MNAR: Missing Not at Random
MI: Multiple Imputation
HOOS: Hip Disability and Osteoarthritis Outcome Score
ADL: Activities of Daily Living
TUG: Timed Up & Go
6 MWT: 6 Minute Walk Test
SD: Standard Deviation
ANCOVA: Analysis of Covariance
ITT: Intention to Treat
CERT: Consensus on Exercise Reporting Template
WMA: World Medical Association
WOMAC: Western Ontario and McMaster Universities Osteoarthritis Index
GDPR: General Data Protection Regulation
ASA: American Society of Anesthesiologists
MRC: Medical Research Council
