# Supplementary material for: The effect of prehabilitation for older patients awaiting total hip replacement. A randomized controlled trial with long-term follow up
Source: BMC Musculoskelet Disord. 2025 Mar 6;26:227. doi: 10.1186/s12891-025-08468-4 (PMC11884013; doi:10.1186/s12891-025-08468-4)
Supplement: Supplementary file 2 — Supplementary Material 2. [file 12891_2025_8468_MOESM2_ESM.docx]

Appendix Table 3 – Per protocol analysis with mean differences between the intervention and control group (95% CI) at different assessment points throughout the study period

| Outcome | Mean | 95% CI |
| --- | --- | --- |
| 40m Fast-Paced Walk Test (m/s) |  |  |
| Post-intervention  6 weeks post-surgery  3 months post-surgery  6 months post-surgery  12 months post-surgery | 0.22*  -0.30  0.12  0.01  0.11 | 0.04, 0.39  -0.21, 0.15  -0.55, 0.30  -0.17, 0.18  -0.06, 0.28 |
| 30s Sit-To-Stand Test (no. of rep.) |  |  |
| Post-intervention  6 weeks post-surgery  3 months post-surgery  6 months post-surgery  12 months post-surgery | 0.48  -0.32  1.81*  1.31  1.89* | -1.34, 2.30  -2.21, 1.57  0.01, 3.60  -0.47, 3.10  0.14, 3.69 |
| Timed Up and Go Test (s) |  |  |
| Post-intervention  6 weeks post-surgery  3 months post-surgery  6 months post-surgery  12 months post-surgery | -0.77  0.89  -0.67  -0.06  -0.54 | -1.87, 0.32  -0.25, 2.03  -1.74, 0.40  -1.13, 0.99  -1.58, 0.50 |
| 6 min Walk Test (m) |  |  |
| Post-intervention  6 weeks post-surgery  3 months post-surgery  6 months post-surgery  12 months post-surgery | 7.79  11.37  35.16  17.28  19.45 | -34.60, 50.18  -31.30, 54.06  -6.73, 77.05  -24.41, 58.98  -21.63, 60.53 |
| Stair Climb Test (s) |  |  |
| Post-intervention  6 weeks post-surgery  3 months post-surgery  6 months post-surgery  12 months post-surgery | -0.80  0.32  -0.71  1.21  -1.03 | -4.46, 2.84  -3.32, 3.97  -4.24, 2.80  -2.45, 4.69  -4.63, 2.58 |
| HOOS Pain (0-100) |  |  |
| Post-intervention  6 weeks post-surgery  3 months post-surgery  6 months post-surgery  12 months post-surgery | 4.02  -0.98  2.63  -0.83  -3.12 | -4.53, 12.58  -9.18, 7.22  -4.85, 10.10  -8.41, 6.75  -10.85, 4.61 |
| HOOS Symptoms (0-100) |  |  |
| Post-intervention  6 weeks post-surgery  3 months post-surgery  6 months post-surgery  12 months post-surgery | 4.32  -2.63  -2.39  -2.80  -4.06 | -4.38, 13.03  -10.79, 5.54  -9.99, 5.21  -10.51, 4.90  -12.12, 4.00 |
| HOOS ADL (0-100) |  |  |
| Post-intervention  6 weeks post-surgery  3 months post-surgery  6 months post-surgery  12 months post-surgery | 4.85  -2.50  -3.33  -3.47  -2.57 | -3.90, 13.59  -10.88, 5.88  -11.12, 4.46  -11.67, 4.73  -10.77, 5.63 |
| HOOS Sports/recreation (0-100) |  |  |
| Post-intervention  6 weeks post-surgery  3 months post-surgery  6 months post-surgery  12 months post-surgery | 8.61  -1.51  3.00  0.86  -11.79 | -4.95, 22.16  -15.28, 12.27  -9.16, 15.17  -11.86, 13.58  -24.37, 0.80 |
| HOOS QoL (0-100) |  |  |
| Post-intervention  6 weeks post-surgery  3 months post-surgery  6 months post-surgery  12 months post-surgery | 11.05*  3.77  -2.42  -3.09  -6.89 | 0.45, 21.64  -6.32, 13.85  -11.92, 7.08  -12.80, 6.62  -16.68, 2.91 |
| EQ-VAS (0-100) |  |  |
| Post-intervention  6 weeks post-surgery  3 months post-surgery  6 months post-surgery  12 months post-surgery | 7.44  -6.27  -3.69  4.39  3.73 | -3.32, 18.19  -16.57, 4.02  -13.32, 5.94  -5.55, 14.32  -6.25, 13.71 |

Differences between randomized groups for continuous outcome variables analyzed by Linear mixed models for repeated measurements, p-values were two-sided and set to a 5% significance level but not presented in the table, *statistical significance between groups p<0.05
CI: confidence interval, HOOS: Hip Disability and Osteoarthritis Outcome Score, ADL: activities of daily living, QOL: quality of life, EQ-VAS: EuroQol Visual Analogue Scale
