# Supplementary material for: The effect of prehabilitation for older patients awaiting total hip replacement. A randomized controlled trial with long-term follow up
Source: BMC Musculoskelet Disord. 2025 Mar 6;26:227. doi: 10.1186/s12891-025-08468-4 (PMC11884013; doi:10.1186/s12891-025-08468-4)
Supplement: Supplementary file 3 — Supplementary Material 3. [file 12891_2025_8468_MOESM3_ESM.docx]

Appendix Table 4 – Descriptive values within groups for primary and secondary outcomes at different assessment points throughout the study period

| Outcome | Intervention | | Control | |
| --- | --- | --- | --- | --- |
|  | n | Mean (SD) | n | Mean (SD) |
| 40m Fast-Paced Walk Test (m/s) |  |  |  |  |
| Baseline  Post-intervention  6 weeks post-surgery  3 months post-surgery  6 months post-surgery  12 months post-surgery | 48  25  22  26  28  23 | 1.21 (0.38)  1.38 (0.37)  1.38 (0.45)  1.52 (0.37)  1.54 (0.39)  1.57 (0.41) | 50  26  16  26  23  24 | 1.29 (0.37)  1.28 (0.34)  1.44 (0.36)  1.48 (0.32)  1.57 (0.28)  1.51 (0.31) |
| 30s Sit-to-Stand Test (no. of rep.) |  |  |  |  |
| Baseline  Post-intervention  6 weeks post-surgery  3 months post-surgery  6 months post-surgery  12 months post-surgery | 48  25  19  26  28  23 | 10.33 (3.71)  11.56 (3.34)  11.16 (3.40)  13.81 (2.63)  14.21 (4.84)  14.22 (3.77) | 50  26  16  26  23  24 | 10.02 (3.64)  10.23 (3.69)  11.75 (2.35)  12.42 (3.34)  13.39 (3.62)  12.75 (3.77) |
| Timed Up and Go Test (s) |  |  |  |  |
| Baseline  Post-intervention  6 weeks post-surgery  3 months post-surgery  6 months post-surgery  12 months post-surgery | 47  25  20  26  28  23 | 11.13 (3.41)  9.80 (3.08)  10.51 (3.82)  8.85 (1.58)  8.73 (1.91)  8.37 (1.78) | 50  26  16  26  23  24 | 11.13 (3.63)  10.44 (2.78)  8.99 (1.76)  8.84 (2.10)  8.32 (1.48)  8.41 (1.43) |
| 6 min Walk Test (m) |  |  |  |  |
| Baseline  Post-intervention  6 weeks post-surgery  3 months post-surgery  6 months post-surgery  12 months post-surgery | 48  25  22  26  28  23 | 353.04 (104.98)  374.48 (101.81)  391.40 (121.64)  430.30 (104.74)  448.92 (105.06)  449.34 (105.57) | 50  26  16  26  23  24 | 353.23 (104.12)  343.50 (98.03)  375.68 (105.70)  419.07 (83.92)  425.26 (109.78)  437.70 (83.32) |
| Stair Climb Test (s) |  |  |  |  |
| Baseline  Post-intervention  6 weeks post-surgery  3 months post-surgery  6 months post-surgery  12 months post-surgery | 47  25  21  26  28  22 | 18.51 (8.41)  16.17 (9.89)  15.68 (10.57)  12.36 (5.70)  12.79 (7.13)  11.95 (5.76) | 48  25  16  25  22  24 | 19.99 (11.78)  17.97 (8.60)  14.21 (4.21)  13.44 (8.21)  11.19 (3.79)  11.71 (4.90) |
| HOOS Pain (0-100) |  |  |  |  |
| Baseline  Post-intervention  6 weeks post-surgery  3 months post-surgery  6 months post-surgery  12 months post-surgery | 45  25  27  34  31  25 | 41.33 (12.05)  46.40 (15.58)  78.61 (14.76)  84.49 (17.79)  88.39 (14.62)  89.70 (12.95) | 47  26  18  28  23  24 | 41.65 (12.57)  47.21 (11.18)  80.83 (12.97)  87.59 (12.16)  92.50 (10.08)  93.54 (10.95 |
| HOOS Symptoms (0-100) |  |  |  |  |
| Baseline  Post-intervention  6 weeks post-surgery  3 months post-surgery  6 months post-surgery  12 months post-surgery | 45  25  28  34  31  24 | 41.41 (16.72)  46.60 (21.10)  73.75 (14.94)  79.12 (14.89)  80.97 (15.07)  85.21 (16.51) | 49  26  20  28  26  24 | 39.08 (14.67)  44.04 (15.36)  78.25 (13.79)  83.04 (11.89)  84.23 (12.93)  91.46 (10.47) |
| HOOS ADL (0-100) |  |  |  |  |
| Baseline  Post-intervention  6 weeks post-surgery  3 months post-surgery  6 months post-surgery  12 months post-surgery | 44  24  25  34  30  24 | 42.08 (13.41)  47.73 (18.38)  77.88 (14.40)  82.09 (17.00)  86.13 (15.02)  88.48 (13.84) | 46  27  17  26  22  24 | 44.53 (14.71)  47.49 (14.03)  80.28 (10.21)  87.10 (11.84)  88.70 (12.87)  92.46 (9.84) |
| HOOS Sports/recreation (0-100) |  |  |  |  |
| Baseline  Post-intervention  6 weeks post-surgery  3 months post-surgery  6 months post-surgery  12 months post-surgery | 44  24  25  33  30  23 | 26.85 (19.46)  29.17 (16.24)  47.50 (23.38)  60.98 (23.12)  74.17 (23.37)  77.17 (24.17) | 50  23  13  23  21  20 | 27.00 (17.74)  22.23 (13.79)  48.08 (18.81)  65.22 (23.14)  74.11 (27.47)  85.00 (19.49) |
| HOOS QoL (0-100) |  |  |  |  |
| Baseline  Post-intervention  6 weeks post-surgery  3 months post-surgery  6 months post-surgery  12 months post-surgery | 47  25  28  33  30  25 | 27.13 (13.74)  35.00 (16.03)  70.54 (21.16)  76.70 (20.62)  84.58 (16.39)  84.50 (16.44) | 50  27  19  29  26  24 | 26.50 (13.86)  26.16 (12.50)  67.76 (21.67)  82.33 (16.02)  86.06 (14.39)  91.67 (12.03) |
| EQ-VAS (0-100) |  |  |  |  |
| Baseline  Post-intervention  6 weeks post-surgery  3 months post-surgery  6 months post-surgery  12 months post-surgery | 47  25  28  34  32  25 | 56.55 (21.64)  60.00 (16.33)  73.50 (18.41)  74.35 (15.53)  79.19 (13.24)  77.28 (11.68) | 50  27  20  29  26  24 | 54.00 (17.52)  53.41 (17.60)  81.50 (10.65)  77.38 (14.91)  77.77 (18.44)  77.17 (15.90) |

Presented data are mean with standard deviation (SD)

HOOS: Hip Disability and Osteoarthritis Outcome Score, ADL: activities of daily living, QOL: quality of life, EQ-VAS: EuroQol Visual Analogue Scale
