# Supplementary material for: The effect of prehabilitation for older patients awaiting total hip replacement. A randomized controlled trial with long-term follow up
Source: BMC Musculoskelet Disord. 2025 Mar 6;26:227. doi: 10.1186/s12891-025-08468-4 (PMC11884013; doi:10.1186/s12891-025-08468-4)
Supplement: Supplementary file 4 — Supplementary Material 4. [file 12891_2025_8468_MOESM4_ESM.docx]

Appendix Table 5 – Descriptive mean scores for performance-based outcome measures at different assessment points throughout the study period, stratified by sex

| Baseline | | |
| --- | --- | --- |
| 40m Fast-Paced Walk Test (m/s)   - Male - Female | Intervention (n=48)   - 1.25 - 1.19 | Control (n=50)   - 1.48 - 1.23 |
| 30s Sit-To-Stand (reps)   - Male - Female | Intervention (n=48)   - 10.43 - 10.26 | Control (n=50)   - 11.92 - 9.35 |
| Timed Up and Go (s)   - Male - Female | Intervention (n=47)   - 10.68 - 11.49 | Control (n=50)   - 10.22 - 11.45 |
| 6 Minute Walk Test (m)   - Male - Female | Intervention (n=48)   - 369.02 - 340.11 | Control (n=50)   - 375.23 - 344.14 |
| Stair Climb Test (s)   - Male - Female | Intervention (n=47)   - 16.72 - 19.94 | Control (n=48)   - 15.57 - 21.73 |
| Post-intervention | | |
| 40m Fast-Paced Walk Test (m/s)   - Male - Female | Intervention (n=25)   - 1.44 - 1.32 | Control (n=26)   - 1.45 - 1.20 |
| 30s Sit-To-Stand (reps)   - Male - Female | Intervention (n=25)   - 12.08 - 11.00 | Control (n=26)   - 12.38 - 9.28 |
| Timed Up and Go (s)   - Male - Female | Intervention (n=25)   - 8.96 - 10.71 | Control (n=26)   - 9.27 - 10.69 |
| 6 Minute Walk Test (m)   - Male - Female | Intervention (n=25)   - 384.30 - 363.83 | Control (n=26)   - 368.00 - 332.61 |
| Stair Climb Test (s)   - Male - Female | Intervention (n=25)   - 13.40 - 19.34 | Control (n=25)   - 14.30 - 19.69 |
| 6 weeks post-surgery | | |
| 40m Fast-Paced Walk Test (m/s)   - Male - Female | Intervention (n=22)   - 1.57 - 1.22 | Control (n=16)   - 1.63 - 1.38 |
| 30s Sit-To-Stand (reps)   - Male - Female | Intervention (n=19)   - 12.57 - 10.33 | Control (n=16)   - 10.75 - 12.08 |
| Timed Up and Go (s)   - Male - Female | Intervention (n=20)   - 9.29 - 11.32 | Control (n=16)   - 8.01 - 9.32 |
| 6 Minute Walk Test (m)   - Male - Female | Intervention (n=22)   - 432.70 - 357.00 | Control (n=16)   - 427.50 - 358.41 |
| Stair Climb Test (s)   - Male - Female | Intervention (n=21)   - 10.79 - 19.34 | Control (n=16)   - 11.00 - 15.28 |
| 3 months post-surgery | | |
| 40m Fast-Paced Walk Test (m/s)   - Male - Female | Intervention (n=26)   - 1.67 - 1.40 | Control (n=26)   - 1.75 - 1.38 |
| 30s Sit-To-Stand (reps)   - Male - Female | Intervention (n=26)   - 14.08 - 13.57 | Control (n=26)   - 13.71 - 12.00 |
| Timed Up and Go (s)   - Male - Female | Intervention (n=26)   - 8.65 - 9.02 | Control (n=26)   - 7.72 - 9.41 |
| 6 Minute Walk Test (m)   - Male - Female | Intervention (n=26)   - 450.83 - 412.71 | Control (n=26)   - 480.42 - 394.47 |
| Stair Climb Test (s)   - Male - Female | Intervention (n=26)   - 10.92 - 13.59 | Control (n=25)   - 9.49 - 14.79 |
| 6 months post-surgery | | |
| 40m Fast-Paced Walk Test (m/s)   - Male - Female | Intervention (n=28)   - 1.79 - 1.38 | Control (n=23)   - 1.75 - 1.49 |
| 30s Sit-To-Stand (reps)   - Male - Female | Intervention (n=28)   - 15.18 - 13.59 | Control (n=23)   - 13.71 - 13.25 |
| Timed Up and Go (s)   - Male - Female | Intervention (n=28)   - 7.85 - 9.29 | Control (n=23)   - 7.72 - 8.58 |
| 6 Minute Walk Test (m)   - Male - Female | Intervention (n=28)   - 493.09 - 420.35 | Control (n=23)   - 480.42 - 401.12 |
| Stair Climb Test (s)   - Male - Female | Intervention (n=28)   - 9.58 - 14.87 | Control (n=22)   - 9.49 - 11.99 |
| 12 months post-surgery | | |
| 40m Fast-Paced Walk Test (m/s)   - Male - Female | Intervention (n=23)   - 1.88 - 1.44 | Control (n=24)   - 1.73 - 1.46 |
| 30s Sit-To-Stand (reps)   - Male - Female | Intervention (n=23)   - 16.29 - 13.31 | Control (n=24)   - 11.60 - 13.05 |
| Timed Up and Go (s)   - Male - Female | Intervention (n=23)   - 7.62 - 8.70 | Control (n=24)   - 7.35 - 8.69 |
| 6 Minute Walk Test (m)   - Male - Female | Intervention (n=23)   - 508.85 - 423.31 | Control (n=24)   - 512.80 - 417.94 |
| Stair Climb Test (s)   - Male - Female | Intervention (n=22)   - 8.76 - 13.15 | Control (n=24)   - 9.37 - 12.32 |
