# Supplementary material for: The effect of prehabilitation for older patients awaiting total hip replacement. A randomized controlled trial with long-term follow up
Source: BMC Musculoskelet Disord. 2025 Mar 6;26:227. doi: 10.1186/s12891-025-08468-4 (PMC11884013; doi:10.1186/s12891-025-08468-4)
Supplement: Supplementary file 5 — Supplementary Material 5. [file 12891_2025_8468_MOESM5_ESM.docx]

Appendix Table 6. Frequencies of aid usage during physical performance testing in the intervention (I) and control (C) group at different assessment points throughout the study period, n (%)

|  | 40m Fast-Paced Walk Test | 30s Sit-to-Stand Test | Timed Up and Go Test | 6 min Walk Test | Stair Climb Test |
| --- | --- | --- | --- | --- | --- |
| Baseline | I: 6 (12.5%) C: 11 (22%) | I: 7 (14.6%) C: 10 (20%) | I: 3 (6.3%) C: 9 (18%) | I: 7 (14.6%) C: 12 (24%) | I: 34 (70.8%) C: 35 (70%) |
| Post-intervention | I: 2 (4.2%) C: 6 (12%) | I: 3 (6.3%) C: 2 (4%) | I:1 (2.1%)* C: 6 (12%) | I: 4 (8.3%) C: 7 (14%) | I: 16 (33.3%) C: 19 (38%) |
| 6 weeks post-surgery | I: 6 (12.5%) C: 2 (4%) | I: 2 (4.2%) C: 1 (2%) | I: 4 (8.3%) C: 0 (0%) | I: 8 (16.7%) C: 3 (6%) | I: 17 (35.4%) C: 12 (24%) |
| 3 months post-surgery | I: 1 (2.1%) C: 2 (4%) | I: 0 (0%) C: 0 (0%) | I: 0 (0%)  C: 1 (2%) | I: 1 (2.1%) C: 3 (6%) | I: 21 (43.8%) C: 15 (30%) |
| 6 months post-surgery | I: 2(4.2%) C: 0 (0%) | I: 0 (0%) C: 0 (0%) | I: 0 (0%) C: 0 (0%) | I: 2(4.2%) C: 1 (2%) | I: 20 (41.7%) C: 16 32%) |
| 12 months post-surgery | I: 2 (4.2%) C: 2 (4%) | I: 0 (0%) C: 1 (2%) | I: 0 (0%) C: 1 (2%) | I: 3 (6.3%) C: 2 (4%) | I: 20 (41.7%) C: 20 (40%) |

Between-group analysis is assessed with Pearson χ2 tests, *p<0.05.
